# Small Molecules, α-Synuclein Pathology, and the Search for Effective Treatments in Parkinson’s Disease

**DOI:** 10.3390/ijms252011198

**Published:** 2024-10-18

**Authors:** Gian Pietro Sechi, M. Margherita Sechi

**Affiliations:** Department of Medicine, Surgery and Pharmacy, University of Sassari, 07100 Sassari, Italy

**Keywords:** Parkinson’s disease, α-synuclein, intermediary metabolism, ß-hydroxybutyrate, thiamine, small molecules, treatment

## Abstract

Parkinson’s disease (PD) is a progressive age-related neurodegenerative disorder affecting millions of people worldwide. Essentially, it is characterised by selective degeneration of dopamine neurons of the nigro-striatal pathway and intraneuronal aggregation of misfolded α-synuclein with formation of Lewy bodies and Lewy neurites. Moreover, specific small molecules of intermediary metabolism may have a definite pathophysiological role in PD. These include dopamine, levodopa, reduced glutathione, glutathione disulfide/oxidised glutathione, and the micronutrients thiamine and ß-Hydroxybutyrate. Recent research indicates that these small molecules can interact with α-synuclein and regulate its folding and potential aggregation. In this review, we discuss the current knowledge on interactions between α-synuclein and both the small molecules of intermediary metabolism in the brain relevant to PD, and many other natural and synthetic small molecules that regulate α-synuclein aggregation. Additionally, we analyse some of the relevant molecular mechanisms potentially involved. A better understanding of these interactions may have relevance for the development of rational future therapies. In particular, our observations suggest that the micronutrients ß-Hydroxybutyrate and thiamine might have a synergistic therapeutic role in halting or reversing the progression of PD and other neuronal α-synuclein disorders.

## 1. Introduction

Parkinson’s disease (PD) is a progressive age-related neurodegenerative disorder affecting millions of people worldwide, for which, currently, only symptomatic treatment is available. However, this treatment does not affect disease progression [1,2]. Therefore, finding rational, innovative neuroprotective compounds for this disorder represents an important and urgent need. The aetiology of PD is complex and both genetic and environmental factors may contribute [1,2]. Extensive research on PD pathophysiology has demonstrated that definite biochemical pathways are involved in specific neuronal cells and discrete areas of the brain, such as the dopaminergic neurons in substantia nigra pars compacta (SNc) and the nigro-striatal pathway [1,2,3]. Moreover, several specific small molecules (i.e., a molecular weight ≤ 1000 daltons) of intermediary metabolism may have a definite pathophysiological role in PD. The affected metabolic pathways include the catecholaminergic system, the glutathione synthesis pathway, the tryptophan/kynurenine catabolic pathway, polyamine pathway, purine pathway, fatty acid- and beta oxidation, as well as the concentration changes of several amino acids [4,5,6,7,8,9].

Considering the myriad of small molecules potentially involved, and the huge number of experimental and clinical findings documenting the definite pathophysiological and therapeutic relevance of specific small molecules in PD, we have chosen to draw attention especially to the biological action of dopamine, levodopa, reduced glutathione (GSH), glutathione disulfide/oxidised glutathione (GSSG), and the micronutrients thiamine and ß-Hydroxybutyrate [4,5,6]. Recent research indicates that these small molecules can interact with the small protein α-synuclein (α-syn) and regulate its folding and potential aggregation, a likely critical event in PD pathophysiology [10,11,12].

This review article summarises and discusses the recent developments on the relationship between these small molecules and the folding and potential aggregation of α-syn in patients with PD, and analyses some of the main molecular mechanisms potentially involved. Better understanding of these interactions may have relevance for the development of rational future therapies effective in halting the progression of PD and other neuronal α-syn disorders [13]. Notably, several review articles offering new therapeutic insights in different experimental models of PD and into the various clinical phenotypes and different stages of PD, including those focusing on small molecules therapy, have been recently published [11,12,14,15,16]. However, they did not discuss the biological action and possible therapeutic role in halting or reversing the progression of PD of the small molecules of intermediary metabolism examined in this review.

## 2. Brain-Specific Small Molecules in Parkinson’s Disease

Individuals with PD may vary strongly in their clinical manifestations and overall prognosis, suggesting that PD may be divisible into subtypes. A recent published study on newly diagnosed untreated patients with PD, recommended four different subtypes based on both motor and nonmotor signs/symptoms and progression rate [17]. The exact mechanisms underlying the progressive loss of dopamine neurons in the nigro-striatal pathway, and the impairment of other types of neurons in noradrenergic, cholinergic, and serotonergic pathways in PD brain still remains elusive [1,2]. Important scientific advances in α-syn research indicate that the intracellular pathological accumulation and spreading of toxic α-syn aggregates is a likely critical event in PD pathophysiology [10,11,12]. According to the Braak hypothesis [18], in most cases of PD, the onset of α-syn pathological aggregation begins outside the central nervous system, in the olfactory bulb and/or the enteric nervous system, and then spreads retrogradely to the brain, penetrating the cerebral hemispheres via the brain stem and subcortex [18,19]. To date, it is believed that, under pathological conditions, α-syn forms oligomers and fibrils due to instability of the α-syn helical structure, in a multi-step process [20]. Primary nucleation, which is the formation of soluble oligomeric intermediates from small aggregates of unfolded monomers, elongation, which is the process of adding monomers to the ends of existing aggregates, and secondary nucleation in which there is a recruitment of soluble proteins into new aggregates [20].

The α-syn aggregates in neurons, astrocytes and microglia lead to various cellular dysfunctions such as impaired mitochondrial function, pathological oxidative stress, endoplasmic reticulum impairment, disruption in the autophagy-lysosomal pathway, synaptic dysregulation and progressive neuroinflammation until neuronal cell death [20]. Importantly, the spread of pathological α-syn oligomers and fibrils is closely associated with the disease progression, and possibly, the individual variation in clinical manifestations of PD may also depend on the spreading patterns and the affected networks [19,20,21]. PD belongs to a group of diseases called α-synucleinopathies, sharing the pathological accumulation and spreading of toxic α-syn aggregates, which includes dementia with Lewy bodies, PD with dementia, multiple system atrophy and neuroaxonal dystrophy [13,20]. The distinct characteristics of different conformational α-syn strains found in neurons and glia may partly explain the heterogenous nature of α-synucleinopathies [20]. Moreover, studies on familial PD, which accounts for about 3–5% of the sporadic cases [1], have shown that most of the genes involved in PD pathology are related to α-syn synthesis, trafficking, and clearance [20]. α-Syn is encoded by the SNCA gene, and missense point mutations in this gene (e.g., A30P, A53T, E46K, G51D, A53E, and H50Q) increase the potential of α-syn for misfolding and aggregation, and affect the conformation of α-syn fibrils [20,22]. Interestingly, compared to the wild-type protein, these different mutations of α-syn exhibit different ability in promoting oligomerisation, fibrillation, and the formation of the insoluble fibrillar inclusions in cells, thus showing a great difference in their cytotoxicity and seeding capacity (as described in depth in reference [20]).

Importantly, since the first report that mutations in the SNCA gene are involved in the early-onset familial PD, many other genetic mutations have been attributed to PD [1]. These include mutations in LRRK2 (encoding leucine-rich repeat kinase 2) and PRKN (encoding parkin) [1]. Mutations in the LRRK2 gene are the most frequent known cause of late-onset autosomal dominant and sporadic PD, while mutations in the PRKN gene usually lead to a loss of parkin activity and are the most common recessive form of PD, frequently with disease onset before the age of 40 years [1]. Notably, pathologic mutations of the LRRK2 protein induce neuronal degeneration both through a gain of function mechanism and, in part, because of its effect on α-syn [23]. Indeed, PD-associated mutations in LRRK2 contribute to α-syn aggregation, its release from cells, and propagation to other cells [24]. Consequently, therapies targeting LRRK2 may have also a role in treating the neurodegeneration associated with pathological α-syn accumulation [23,24].

Likewise, variation in the concentration/value of several factors in the microenvironment of discrete cerebral areas in PD patients may foster the post-translational pathological assembly and potential aggregation of α-syn. These factors include molecular crowding, the presence of specific metal ions and proteins (e.g., Ca^2+^, Mg^2+^, Fe^2+^; tau protein and amyloid-ß peptide), acidic pH, the excessive generation of ROS, disruption of lipid homeostasis, and the levels of brain-specific molecules of intermediary metabolism such as dopamine, the dopamine metabolite 3,4-dihydroxyphenylacetaldehyde, and glutathione disulfide/oxidised glutathione [6,25,26]. Moreover, definite biochemical reactions, such as phosphorilation and tyrosine nitration of α-syn, may also foster its post-translation pathological assembly and potential aggregation [20,25]. Brain-specific small molecules in PD can be defined as those small molecules with a known, definite and important metabolic role in dopamine neurons of the nigro-striatal pathway or other multiple pathways in the brain related to PD (e.g., the catecholaminergic system and the glutathione synthesis pathway). Importantly, although this definition is suitable for the purpose of the review, it is worth noting that actually no small molecules are really “brain-specific”, indeed all small molecules of intermediary metabolism may be detected both inside and outside the brain, even at very different concentrations, because only ~2% to 6% of all small molecules can cross the blood-brain barrier [27].

The occurrence of a marked decrease of dopamine concentrations in the striatum of patients with PD, shown by Ehringer and Hornykiewicz in 1960 [4], indicated a means by which the nigra cells could be therapeutically supported. In PD patients, this discovery led to the extensive use of levodopa in clinical practice [1]. Levodopa is the precursor to dopamine that, unlike dopamine, can cross the blood-brain barrier [1]. Importantly, this large, neutral aminoacid, still remains the most effective symptomatic treatment for motor symptoms in patients with PD, although it does not affect disease progression [1]. This is because, in most PD patients, motor symptoms become evident when 50–80% of nigral dopaminergic neurons have degenerated, after the occurrence of irreversible structural lesions in these neuronal cells [1].

In 1982, Perry et al. [5] showed a marked decrease of GSH levels in substantia nigra in cases of incidental Lewy body disease (presymptomatic PD) [5]. In particular, the magnitude of reduction in GSH seems to parallel the severity of PD motor symptoms and, in advanced stages, in the nigra, GSH is virtually undetectable [5]. The occurrence of GSH depletion, in tandem with altered iron metabolism in substantia nigra of patients with PD, has been recently replicated in a study by Shukla et al. [28] This evidence suggests a fundamental role of redox dysfunction and specific oxidation reactions in the development and progression of PD. Redox dysfunction in neuronal cells that seems to occur at an early stage of PD, likely in the so-called stage of “reversible biochemical lesions”, when the use of appropriate disease-modifying treatments should be effective in halting the progression of the disorder. Moreover, these findings provide a potential early diagnostic biomarker for PD and suggest a therapeutic role of GSH, its precursors and analogues, or other antioxidants molecules of intermediary metabolism, such as thiamine and ß-Hydroxybutyrate, in PD. Notably, some experimental studies documented that GSH may cross the blood-brain barrier intact by a saturable, low-affinity transport process [29], and in a Sechi et al. study in 1996, the administration of large doses of GSH intravenously to untreated, early PD patients, improved bradykinesia and other PD motor symptoms significantly [30]. In support of this finding, a mild symptomatic effect of lower doses of intravenous GSH on PD motor symptoms in patients not adequately controlled with their current medication was shown by Hauser et al. in 2009, in a randomised controlled trial [31].

Moreover, many experimental studies have documented a definite relationship between dopamine metabolism in neuronal cells of the nigro-striatal pathway and the micronutrients thiamine and ß-Hydroxybutyrate. Indeed, intrastriatal administration of thiamine diphosphate in rats can release a significant amount of dopamine, whereas reduced levels of dopamine in the striatum can occur in thiamine deficiency [32,33]. Furthermore, a significant decrease of CSF-free thiamine levels has been shown in patients with PD, as compared to controls, and the administration of 100/200 mg daily doses of parenteral thiamine in five PD patients has been reported to improve motor symptoms within few days of treatment [34,35]. Similarly, many experimental studies have documented a beneficial effect of ß-Hydroxybutyrate in different models of PD [36]. In particular, the infusion of this ketone body in mice with MPTP-induced parkinsonism rescues mitochondrial respiration, increases ATP production, and mitigates features of PD [36]. These findings fit preliminary clinical data showing that ketogenic diet may be beneficial in PD [37].

## 3. Brain-Specific Small Molecules in Parkinson’s Disease and α-Synuclein Pathology

**Figure 1:**
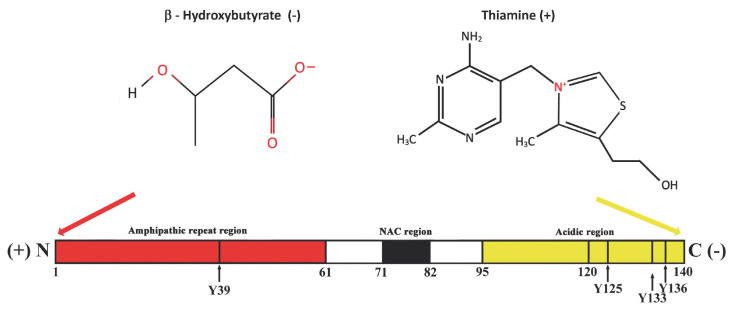
The α-synuclein protein, its three regions, and the potential interaction with ß-Hydroxybutyrate and thiamine.

Parkinson’s disease is associated with the progressive formation of misfolded α-syn and its aggregation both inside and outside the brain [1,2]. In neurons, this pathological misfolding forms aggregates called Lewy bodies and Lewy neurites, chiefly composed of misfolded α-syn [38]. At a biochemical level, pathological oxidative stress, inflammatory, and immune responses may foster the incorrect folding of α-syn, the progressive formation of toxic oligomers and insoluble amyloid fibrils, ultimately causing neuronal loss and this degenerative disorder [39,40]. Alpha-synuclein is a small, soluble, 140 amino acid protein, which is widely expressed in the brain. Its sequence can be divided in three regions: the N-terminal amphipathic region, which contains most of the mutations linked to autosomal dominant early-onset PD, and the repeated KTKEGV sequences that mediate the interaction between α-syn and the surface of acidic lipids. This region maintains the helical structure of α-syn. The central region, encompasses the most hydro-phobic non amyloid-ß component (NAC) of the protein and promotes aggregation. In contrast, the acidic C-terminal portion, which is highly negatively charged, mainly due to the negative charges of Asp and Glu, also contains three of the four Tyr residues (Y125, Y133, Y136) and tends to decrease protein aggregation [41,42] (Figure 1).

In cells, under physiological conditions, soluble α-syn engages in a myriad of interactions with a variety of proteins and small molecules of intermediary metabolism that can affect its correct or incorrect folding and its potential aggregation. Although, current understanding of the mechanisms underlying these interactions remains relatively limited, recent work proposes that the interaction between α-syn and specific, different proteins may lead to the formation of distinct strains, and contribute to the clinical heterogeneity observed among PD patients [43].

Concerning the small molecules of intermediary metabolism previously discussed, a pioneering study in vitro indicated that catecholamines, including dopamine and levodopa could interfere with the aggregation process of α-syn and disaggregate amyloid fibrils [44]. However, these findings have not been replicated in vivo. Instead, the occurrence of an endotoxicity associated with levodopa administration and the formation of dangerous dopamine metabolites (e.g., 3,4 dihydroxyphenylacetaldehyde) seems to occur, which triggers α-syn oligomerisation [45]. Moreover, in animal models of PD, the manipulation of both dopamine levels and α-syn expression in aged mice induces soluble α-syn oligomers and nigro-striatal degeneration [46,47]. Similarly, the amyloid formation of α-syn was significantly facilitated by GSSG. Indeed, while α-syn itself started to form aggregates after 125 h of prolonged lag period, GSSG reduced the lag to around 50 h. Interestingly, GSH did not influence the lag phase, although it increased the final amyloid formation [48]. This is because, under specific conditions, the oxidative stress may favour GSSG formation from GSH [48]. This observation indicates that, in cells, GSH and GSSG contents are finely regulated and raises the issue on how to supplement the GSH content of neurons without also eliciting potential detrimental effects.

The micronutrients, ß-Hydroxybutyrate, a ketone body produced by the liver, and thiamine, the water-soluble B1 vitamin, possess high permeability across the blood-brain barrier (BBB) [49,50]. In particular, for ß-Hydroxybutyrate, this is also related to a specific modulable carrier [49]. Thiamine transport at the BBB, instead, occurs by both passive and active mechanisms, which allows for a rapid increase of brain thiamine concentrations after parenteral administration of the vitamin [50]. These micronutrients are small molecules essential to protecting cells against energy deficit, oxidative stress, neuroinflammation, and cellular death by apoptosis [51,52]. Thus, they may also exert a crucial neuroprotective effect on dopaminergic neurons in PD [53,54]. In particular, a molecular analysis of their chemical characteristics, and of the charge-dependent intermolecular interactions, indicates that these molecules may bind α-syn in specific, different regions favouring the correct folding of the protein and counteracting its pathological aggregation. Indeed, ß-Hydroxybutyrate carries a negative charge and may interact with the positively charged N-terminal amphipathic region of α-syn through the KTKEGV sequences, fostering in this way the maintenance of the helical structure of the protein [42,55,56]. Notably, in *C. elegans* experimental PD-model, treatment with ß-Hydroxybutyrate decreased α-syn aggregation by 35% [57]. Likewise, a study on α-syn fission on a yeast model showed that, in cells, thiamine lowers α-syn concentrations in a dose-dependent manner and, consequently, its potential aggregation [58].

Free thiamine, instead, at physiological pH, carries a positive charge (formal charge, 1+) and may interact with the acidic C-terminal portion of α-syn, which is highly negatively charged, mainly due to the negative charge of Glu and Asp residues [41,42]. Interestingly, a similar mechanism has been also reported in a small thiamin-binding protein from sunflower seeds which contains a large amount of Glu and Asp residues [59].

**Figure 2:**
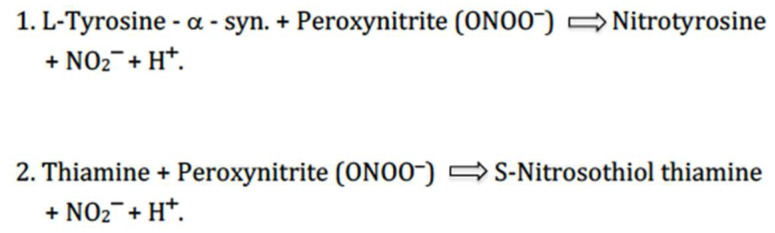
Reactions of Thiamine and Tyrosine residues of α-synuclein with Peroxynitrite. Suggested competing effects of thiamine towards tyrosine residues of α-synuclein protein for peroxynitrite binding: reduction and blocking of the oxidant action of per-oxynitrite on tyrosine residues of α-synuclein, due to the greater affinity of peroxyni-trite (formal charge, 1−) for thiamine (formal charge, 1+) compared with tyrosine (formal charge, 0). For reactions 1 and 2, see also reference [52].

Moreover, in this region, during neuro-oxidative stress, thiamine may bind the negatively charged peroxynitrite anions (ONOO^−^) (formal charge, 1−) and protect the four Tyr residues of α-syn from oxidation, which play a crucial role in decreasing protein aggregation [42,52] (Figure 2).

Notably, increased levels of nitrotyrosine, a specific marker of ONOO^--^ attack on tyrosine residues of proteins, were previously demonstrated in many neurodegenerative disorders including PD [60]. Also, it is worth nothing that, amongst other modifications, the reaction between tyrosine and peroxynitrite at concentrations ≤5 µM can give rise to covalent links between adjacent tyrosine residues known as dityrosine cross-linking, which could play an important role in the pathological assembly of α-syn in PD [61,62,63,64]. Importantly, under specific experimental conditions, the formation of nitrotyrosine and dityrosine from the reaction between tyrosine and peroxynitrite is inhibited by thiamine [52,65]. Taken together these findings suggest that nitrotyrosine and dityrosine could serve as biomarkers of chronic oxidative damage of proteins in PD, and also indicate specific reactions/pathways that may serve as targets for potential disease-modifying treatments in PD such as thiamine or other, positively charged, antioxidants compounds (e.g., methylene blue). Notably, considering the early, very fast nitration of α-syn tyrosine residues by peroxynitrite in PD [66], thiamine and related compounds are likely to affect the initialisation of monomeric α-syn aggregation, preventing the primary nucleation (i.e., the formation of droplets with high concentrations of α-syn) and the pathological elongation processes of the protein [67,68].

## 4. Other Small Molecules That Target α-Synuclein Aggregation

In addition to the few, brain-specific small molecules of intermediary metabolism related to PD previously discussed, many other natural or synthetic organic small molecules have been studied in vitro and in vivo experiments in order to gain a better understanding of α-syn aggregation as a possible therapeutic target for PD [8,9,69]. These organic compounds may interfere with the regulation of many biological processes of intermediary metabolism in cells, including the mechanisms that modulate α-syn content and conformation. In particular, the main mechanisms of action involved for different small molecules include:

(1) The transcriptional regulation of α-syn which may reduce the cellular expression of the protein [70,71]. A well-studied compound is salbutamol, a brain-penetrant asthma medication, ß2-adrenoreceptor ligand, which modulates SNCA transcription through histone 3 lysine 27 acetylation of its promoters and enhancers [71]. Another similar compound is clenbuterol [70,71].
(2) The translation inhibition of α-syn which reduces the cellular expression of the protein. Using sequence-based design, the small molecule synucleozid, a potent inhibitor of the SNCA mRNA that encodes α-syn protein has been identified. It selectively targets the α-syn mRNA 5′ UTR at a specific IRE site, decreasing the amount of SNCA mRNA loaded into polysomes, thus inhibiting SNCA translation and lowering α-syn protein levels in cells [72]. Another compound that reduces α-syn protein translation is posiphen, the (+) enantiomer of the cholinesterase inhibitor phenserine. However, its exact mechanism of action is unknown [73]. Currently, there is a safety and tolerability study of posiphen in patients with PD.
(3) The increased intracellular clearance of α-syn via activation of either the ubiquitin-proteasome system (for normal monomeric α-syn) [74,75], or of the authophagy/lysosomal pathway (for aggregated α-syn) [76,77]. Evaluated compounds include the natural alkaloids harmine and licorine. Additionally, rapamycin, a stimulator of autophagy which increases the clearance of α-syn [74,75,76,77].
(4) The inhibition of the aggregation, either of monomeric α-syn or of the aggregated α-syn, to prevent further elongation of α-syn fibrils [10,78,79]. Several natural and synthetic small molecules have been studied: polyphenols such as curcumin, epigallocatechin-3-gallate, caffeic acid; the human Rho kinase inhibitor fasudil; the anti-microbials squalamine and ceftriaxone; the selective tyrosine kinase inhibitor nilotinib; the synthetic molecules CLR01, NPT100-18A, Anle138 B, 03A10 and Compound C [10,78,79].
(5) The stabilisation of the physiological conformation of α-syn [71]. The synthetic compound NPT200-11, recently studied in a small randomised, double-blind clinical trial [71].
(6) The prevention of α-syn spread by inhibiting uptake from the neighbouring cells and the fostering of α-syn extracellular clearance mechanisms [71,80,81]. The NMDA receptor antagonists AP5 and memantine, and the AMPA receptor antagonist perampanel are being investigated for their ability to also block α-syn synaptic transmission and decrease the amount of this protein in the extracellular space [80,81].

In recent years, there has been a marked increase in the number of small molecules studied to target α-syn aggregation as a potential therapeutic strategy for PD [8,79] (Table 1). These include natural and synthetic compounds developed by the repositioning of already approved molecules to develop new treatments for different diseases, the analysis of the polyphenol-based scaffolds, the use of a rational design considering specific regions or conformations of a protein, the high-throughput screening of large libraries of compounds, and the use of structure-based strategies using recent advances in structural analyses of in vitro formed and patient-derived α-syn fibrils [79,82,83].

Among the several small molecules identified as having definite anti-aggregational properties toward α-syn, the most relevant molecules include the repositioned drugs ceftriaxone, vancomycin, doxycycline, squalamine and its analogue trodusquemine (antibiotics/anti-microbials) [7,79,84,85,86,87,88]; fasudil (Rho kinase inhibitor/cerebral vasospasm and glaucoma) [87]; clenbuterol and salbutamol (ß2-adrenergic receptor agonists/treatment of asthma) [73,88]; methylene blue/methylthioninium and its reduced stable form leuco-methylthioninium bis(hydromethanesulfonate) (oxidised phenothiazine/antimalarial agent, powerful antioxidant) [89,90]. Additionally, the natural polyphenols curcumin, myricetin, ferulic acid, caffeic acid, gallic acid and ellagic acid [79,91,92,93,94]. Notably, structure-activity relationship analysis of several phenolic compounds indicated that the phenyl group alone does not prevent fibril formation [95]. The inhibitory potential on α-syn aggregation seems to be mainly related to the number, position, and conjugation of the hydroxyl groups at the benzoic acid scaffold [95]. Small molecules developed by a rational design, instead, led to the synthesis of many compounds, such as CLR01, CLR03, NPT100-18A, NPT200-11, which induce, in in vitro and in vivo experimental studies, a reduction of α-syn aggregation and the disassembly of preformed fibrils, possibly by the formation of off-pathway oligomers [96,97]. Notably, a clinical phase 1 study on safety and tolerability of NPT200-11 was completed, but the data have not been published [79]. Several other small molecules have been identified through high-throughput screening and structure-based strategies for drug discovery [79,97]. The best clinical drug candidates against α-syn aggregation include the synthetic molecule anle138b, which has shown high affinity towards oligomers of α-syn thus inhibiting further amyloid aggregation [79,98]. Interestingly, the oral administration of this molecule in different PD mice models ameliorated motor symptoms and survival [99,100,101]. Because the therapeutic effect was also evident in late-stage rodents, the possibility that this molecule could also be effective in the advanced stages of PD, has been suggested [102]. Also of note, recent in vitro studies, by using structure-based strategies, report the discovery of new synthetic small molecules that specifically prevent fibril elongation upon binding to fibril ends, or to the surface of the amyloid fibrils, thus inhibiting the formation of α-syn aggregates [103,104]. These molecules seem to be highly selective for the misfolded α-syn, nontoxic, and active in cells in small concentrations [103,104]. Importantly, most of the small molecules discussed in this study have shown a definite antifibrillogenic activity in various neurodegenerative diseases related to the misfolding of different specific proteins. For instance, several studies have documented a beneficial effect of ceftriaxone on the misfolding of glial fibrillary acidic protein (GFAP) in in vitro and in vivo models of Alexander’s disease (AxD), a genetic astrogliopathy [105,106], and on the misfolding of α-syn in dopaminergic neurons in experimental models of PD and in dementia with Lewy bodies [7,107,108]. Likewise, the natural polyphenolic compound curcumin has the ability to inhibit GFAP misfolding in an in vitro model of AxD [10], α-syn misfolding in experimental models of PD [109,110,111], and amyloid beta oligomers formation and interactions with anionic membranes in experimental models of Alzheimer’s disease [112,113]. The synthetic compound, CLR01, instead, causes a reduction of α-syn aggregation and the disassembly of preformed fibrils in in vitro and in vivo models of PD and other synucleinopathies such as dementia with Lewy bodies and multiple system atrophy [96,114,115].

Also, in experimental models in vitro, CLR01 has shown an inhibitory potential in the aggregation process of different amyloid proteins, such as Alzheimer’s Aß42/Aß40 peptides and tau protein, cellular prion protein and transthyretin related to systemic amyloidosis [116,117]. The evidence that the same compound may have a beneficial effect on the pathological misfolding of different proteins in different neurodegenerative diseases, and thus in several kinds of cells and neuronal pathways, supports the possibility that similar pathophysiological mechanisms may underlie abnormal protein aggregation in these disorders. In particular, it is known that an abnormal accumulation of misfolded proteins in cells is strictly related to transcriptional regulation of the protein that may reduce or increase its cellular expression, oxidative stress, mitochondrial dysfunction, cellular energy failure, and inflammation [116]. Thus, multi-target bioactive compounds, such as ceftriaxone, curcumin and CLR01 [7,54,111,114], which can attenuate oxidative stress, neuroinflammation, restore mitochondrial function and regulate genetic transcription are plausible, rational clinical drug candidates against many different neurodegenerative diseases. Recent clinical rials for PD involving small molecules as potential therapeutic agents that may modify disease course are reported in Table 2.

## 5. Rationale for New Treatments Effective in Halting or Reversing the Progression of Parkinson’s Disease

**Figure 3:**
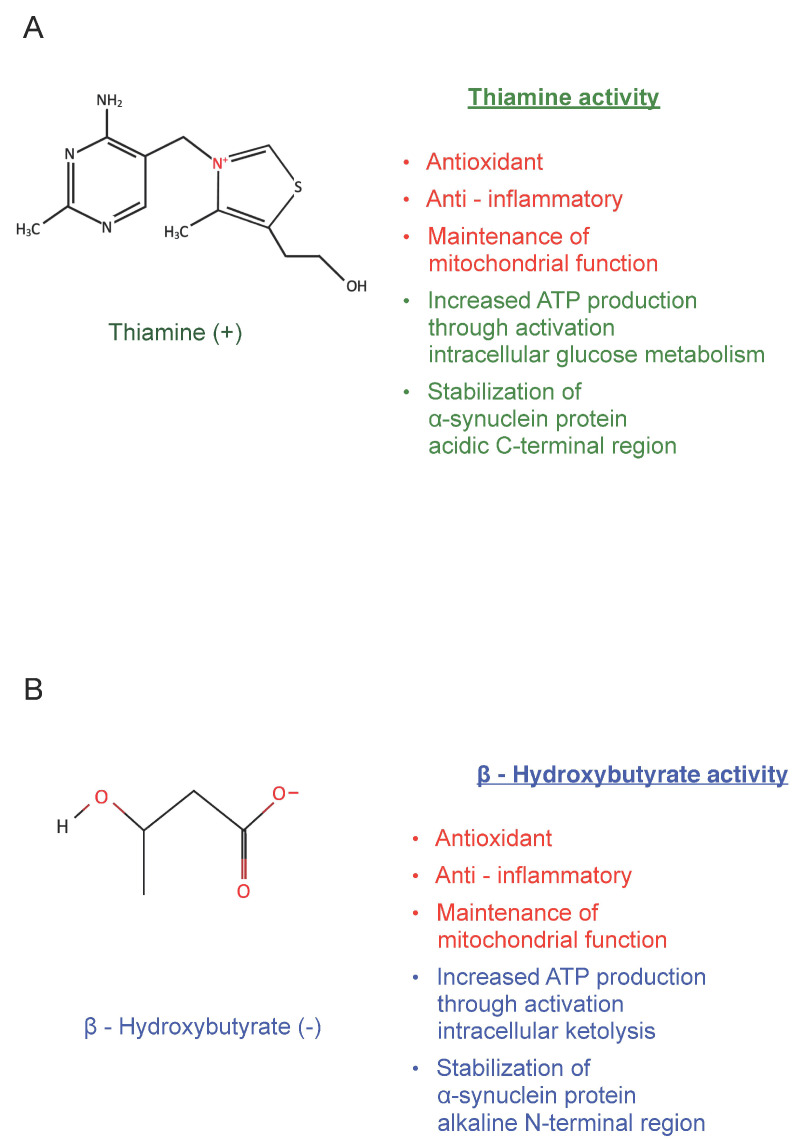
Main biological mechanisms likely to be involved in the therapeutic multi-target mechanism of action of thiamine (**A**) and ß-Hydroxybutyrate (**B**) in halting or reversing the progression of Parkinson’s disease and other neuronal α-synuclein disorders.

In general, the treatment of a disease is based on the known etiopathogenesis, and it will be more effective the more it promptly corrects the early anomalous biological determinants of the disorder. In particular, the known pathophysiological determinants involved in PD include the interaction of specific genetic patterns, chronic oxidative stress, inflammatory and immune responses that may foster the incorrect folding of α-syn and its anomalous aggregation. Moreover, the peculiar, elevated metabolic activity of the dopaminergic neurons in pars compacta of the substantia nigra, directly related to the morphological characteristics of these neurons, which have large size, a very high number of projections and connections into the striatum, and a calcium-dependent, autonomous pacemaking activity that needs one molecule of ATP to pump one molecule of Ca^2+^ into cellular channels to sustain physiological electrochemical gradients [129,130,131,132]. In comparison, a neuron which fires sodium-based action potentials needs only one molecule of ATP to pump into cellular channels three Na^+^ ions and two K^+^ ions [129,130,131,132,133]. Thus, mitochondrial bioenergetic requirements are significantly more elevated in calcium-based action potentials neurons, compared with sodium-based action potentials neurons. As a result, the peculiar morphological and electrophysiological properties of the dopaminergic neurons in the pars compacta of the substantia nigra make them particularly vulnerable to oxidative stress and to biochemical or structural cellular damage [107,108,109,110,111]. In PD patients, considering the likely interaction of many different pathophysiological mechanisms, the use of compounds with definite multi-target activity should be highly promising. In particular, among the brain-specific small molecules of intermediary metabolism in PD previously discussed, reduced glutathione and the micronutrients ß-Hydroxybutyrate and thiamine seem to have these characteristics [30,36,52,54]. Indeed, all three of these compounds can target multiple mechanisms such as oxidative stress, inflammation, mitochondrial damage, and cellular apoptosis. Moreover, thiamine and ß-Hydroxybutyrate exert fundamental roles in energy metabolism. Thiamine via activation of the intracellular glucose metabolism in the cytoplasm and the mitochondria through thiamine pyrophosphate, and ß-Hydroxybutyrate via activation of the ketolytic pathway. Thus, these micronutrients may replenish, in dopaminergic neurons, ATP stores when depleted and protect them against oxidative damage [30,36,54,134]. Also, these micronutrients may interact directly with α-syn in specific, different regions: Hydroxybutyrate with the positively charged N-terminal amphipathic region of α-syn, while thiamine with the acidic C-terminal portion of α-syn [39,42] (Figure 3).

## 6. Conclusions

These observations highlight the relevance of definite small molecules of intermediary metabolism in the pathophysiology of PD. Moreover, the early fundamental role of redox dysfunction and specific oxidative and nitrative α -syn modifications in the development and progression of PD. In addition, they suggest that the micronutrients ß-Hydroxybutyrate and thiamine, that penetrate the blood-brain barrier well and are commercially available, might have a definite therapeutic role in halting or reversing the progression of PD, in particular if used in combination, at an early stage of the disease, when still-reversible biochemical lesions are present in the brain. Indeed, ß-Hydroxybutyrate and thiamine both have antioxidant and anti-inflammatory properties. Moreover, given in combination, they should stabilise two different regions of α-syn protein and, at the same time, through activation of different biochemical pathways involved in energy production (i.e., intracellular glucose and ketone body metabolism) sustain the higher baseline requirements for ATP production in specific dopaminergic neurons.

## Figures and Tables

**Table 1:** Relevant natural and synthetic small molecules targeting α-synuclein folding and aggregation, studied by different methods [56,58,65,74,83].

**1. Repositioning of drugs already approved for different diseases.**
Antibiotics used as anti-microbials: ceftriaxone, vancomycin, doxycycline, squalamine trodusquemine.
Rho kinase inhibitors used for glaucoma and vasospasm: fasudil.ß-2 adrenergic receptor agonists used for asthma: clenbutarol, salbutamol.
Oxidised phenotiazines used as antimalarial agents: methylene blue.
**2. Natural polyphenols. Analysis of the polyphenol-based scaffolds.**
Curcumin, baicalein, myricetin, ferulic acid, caffeic acid, gallic acid, ellagic acid.
**3. Synthetic small molecules developed by rational design.**
CLR01, CLR03, NPT 100-18A, NPT-200-11.
**4. The high-throughput screening of large libraries of compounds.**
Ceftriaxone, Anle138b, SC-D, ZPD-2, ZPDm.
**5. Small molecules identified through structure-based analysis of α-synuclein fibrils.**
PDB.2N0A, Compound C.

**Table 2:** Recent clinical trials for Parkinson’s disease involving small molecules as potential therapeutic agents that may modify disease course.

Agents	Mechanism of Action	Phase Development	Status/Findings
Deferiprone	Iron chelator. Interferes with α-syn aggregation.	Pilot study	Improved motor scores. UPDRS. (Devos, 2014) [118].
		Phase 2/3 NCT00943748	Improved motor scores. UPDRS. (Grolez, 2015) [119]
		Phase 2 NCT01539837	Improvement trend in motor scores. (Martin-Bastida, 2017) [120]
		Phase 3 NCT02655315	Therapy leads to PD worsening. (Devos, NEJM, 2022) [121]
Squalamine phosphate/ENT-01	Interferes with α-syn aggregation by displacing α-syn from membranes.	Phase 2 NCT03047629	Improvement in constipation. (Hauser, 2019) [122]
		Phase 2b NCT03781791	Safe. Improvement in constipation. (Camilleri, 2022) [123]
Buntanetap/Posiphen (ANVS 401)	Reduces α-syn protein translation	Phase 3 NCT05357989	Improvement in motor, nonmotor, cognitive symptoms. (Annovis Bio, New release, 2024) [124]
Anle 138b	Inhibits α-syn oligomer formation	Phase 1 NCT04208152	In healthy volunteers excellent safety, tolerability at all dose-plasma levels [125]
		Phase 2 NCT04685265	Good safety, tolerability confirmed in PD patients (Levin, 2023; Mov Disord) [125]
Nilotinib	c-Abl inhibitor enhances autophagic clearance of α-syn	Phase 2 NCT03205488	Acceptable safety/tolerab. Low brain penetration. No biomarkers effect. No efficacy. (Simuni, 2021, JAMA Neurol) [126]
YTX-7739	Inhibits stearoyl-CoA desaturase, promotes smaller α-syn aggregates	Phase 1b Trial NL 9172	Well tolerated in PD patients. Mild/moderate adverse events (Press Release, 2021, NL) [127]
NPT200-11	Interferes with α-syn aggregation, displacing α-syn from membranes	Phase 1 NCT02606682	In healthy volunteers Data not published. (Journal of Parkinson’s Disease 13; 4:2023) [128]

## Abbreviations

α-syn = α-synuclein; AMPA = α-amino-3-hydroxy-5-methyl-4-isoxazolepropionic acid; Asp = aspartic acid; ATP = adenosine triphosphate; AxD = Alexander’s. disease; BBB = blood-brain barrier; c-Abl = tyrosine kinase Abelson; CSF = cerebrospinal fluid; GFAP = glial fibrillary acidic protein; Glu = glutamic acid; GSH = reduced glutathione; GSSG = glutathione disulfide/oxidised glutathione; IRE = iron-responsive element; LRRK2 = leucine-rich repeat kinase 2; MPTP = 1-methyl-4-phenyl-1,2,3,6-tetra-hydropyridine; mRNA = messenger RNA; NCT = number clinical trial; NMDA = N-methyl-D-aspartate; PD = Parkinson’s disease; ROS = reactive oxygen species; SNc = Substantia Nigra pars compacta; SNCA = α-syn gene; UTR = untranslated region; yrs = years.
